# Linear headache: clinical characteristics of eight new cases

**DOI:** 10.1186/s40064-016-1991-1

**Published:** 2016-03-18

**Authors:** Ya-Nan Lu, Qing-Qing Pan, Jie-Feng Pan, Lei Wang, Yun-Yun Lu, Liang-Hui Hu, Yu Wang

**Affiliations:** Department of Neurology, Epilepsy and Headache Group, the First Affiliated Hospital of Anhui Medical University, Jixi Road 218, Hefei, 230022 China; Department of Cardiology, the Second Division Korla Hospital of Xinjiang Production and Construction Corps, Jiaotong Road, Korla, 841000 Xinjiang China; Department of Neurology, the Second Division Korla Hospital of Xinjiang Production and Construction Corps, Jiaotong Road, Korla, 841000 Xinjiang China

**Keywords:** Linear headache, Epicrania fugax, Cranial neuralgia, Paroxysmal hemicrania, Nummular headache, Cervicogenic headache, Migraine

## Abstract

**Background:**

Linear headache (LH) has recently been described as a paroxysmal or continuous fixed head pain restricted in a linear trajectory of 5–10 mm in width, linking one endpoint in occipital or occipitocervical region with another endpoint in ipsilateral nasion or forehead region. For some patients, this headache had some features resembling migraine without aura.

**Methods:**

We made a prospective search of patients presenting with a clinical picture comprised under the heading of LH and we have accessed eight new cases. A detailed clinical feature of the headache was obtained in all cases to differentiate with cranial neuralgia, paroxysmal hemicrania, cervicogenic headache, nummular headache and migraine.

**Results:**

The eight LH patients complained of a recurrent moderate to severe, distending, pulsating, or pressure-like pain within a strictly unilateral line-shaped area. The headache duration would be ranged from 1 h to 2 days or persistent for 1–6 months with recurrent worsening of headaches. For some patients, this headache had couple of features similar to that of migraine pattern, such as accompaniments of nausea, vomiting, and phonophobia, diziness, triggering factors of noise, bright night, resting after physical activity, fatigue, menstruation, and response to anti-migraine therapy.

**Conclusions:**

This description reinforces the proposal of LH as a new headache syndrome or a new variant of a previously known headache syndrome, probably of migraine.

## Background

Linear headache (LH) may represent a new headache syndrome or a variant of migraine as proposed recently based on the clinical features of twelve patients who presented with a paroxysmal or chronic fixed head pain restricted in a linear trajectory linking the occipital or occipitocervical region with the ipsilateral nasion or forehead region (Wang et al. 2014b). In some patients, this head pain was accompanied with nausea, vomitting and dizziness. The attacks were spontaneous in most patients, but could also be triggered by noise, stress, staying up late, or resting after physical activity in some other patients. The paroxysms lasted for hours to days in most patients, but for minutes in some rare patients, and their character was described as pulsating, distending or pressure-like. Pain intensity was usually moderate or severe. The frequency of attacks was variable, from one attack per day to five or six attacks per year. Some patients had long-term remission course, while others experienced no remission. The patients’s head pain improved with preventive medications, such as flunarizine, sodium valproate and amitriptyline but not to carbamazepine or oxcarbazepine (Wang et al. 2014b). Since the first description of LH case series, only one more case of LH-like head pain has been reported but it was associated with epicrania fugax (EF) (Pareja and Bandres 2015). Here we describe eight new cases with the same clinical features and their therapeutic responses to different medications.

## Methods

After the publication of the first series of LH patients in June 2014 (Wang et al. 2014b), we started to make a prospective search of new patients presenting with a clinical picture comprised under the heading of LH. Specifically, we aimed to record all patients complaining of fixed pain circumscribed in a linear trajectory bridging the posterior and anterior cephalic regions, at the outpatient office of our Neurology department. Whenever we encountered a patient whose clinical presentations could fulfill the criteria for LH, all the clinical data including clinical presentations, neurological examinations, supplemental examinations and following up for therapeutic responses were registered systematically. This study started from April 2014 when the first report of LH case series was submitted to Dec 2015 when the current report was prepared for submission.

A detailed history was obtained in all cases and a special attention was paid to medical history of head trauma or macroscopic changes of the scalp. Patients were asked to point out the exact location of the pain-affecting area and to delineate it in size and shape. The pain characteristics were carefully assessed, including the spatial features (trajectory distribution), the temporal features (duration and frequency), pain character and intensity at baseline and during exacerbations, and pain accompaniments. The presence of any triggering or facilitating factors or interictal symptoms was also evaluated. A complete physical and neurological examination was performed in all cases. Inspection, palpation and sensory examination of the affecting area, as well as palpation of the supraorbital, infraorbital, minor occipital and greater occipital nerves were included in the examination. Routine blood work-up with erythrocyte sedimentation rate, computed tomography (CT) or magnetic resonance imaging (MRI) of the head were performed in all cases to exclude any underlying disease. Cervical vertebra MRI or X-ray was also taken in all patients to check out the lesions which may cause cervicogenic headache (CEH) whose pain maps in very rare cases were described as linear area similar to that in LH (Cooper et al. 2007).

The cervical flexion–rotation test (FRT), which has been proven to be a valid and reliable test of upper cervical movement impairment associated with CEH (Ogince et al. 2007; Hall et al. 2008, 2010a, b; Bravo Petersen and Vardaxis 2015), was specifically conducted to exclude the potential CEH as suggested in a reader’s comment on the first publication of LH case series (Kinney 2014). The manipulation of FRT was adapted from Hall’s method (Hall et al. 2010b). Briefly, the patient was relaxed in supine and the cervical spine was pre-positioned in maximal end range flexion, then the head was passively rotated to the left and the right. End of the head rotation range was determined either by firm resistance encountered by the examiner or by the onset of pain reported by the patient, whichever came first. A compass attached to the apex of the head by straps was used to record the rotation range to determine whether the FRT was positive [range reduced more than 10° from the normal range of 44° (Hall and Robinson 2004; Ogince et al. 2007)] or negative. Diagnosis of other concomitant headache syndromes was based on the 3rd Edition of The International Headache Classification (ICHD-3) criteria (IHS 2013).

All patients accepted treatments. Three of them received flunarizine alone in dose of 5 mg twice a day, two of them tried successively flunarizine 5 mg twice a day and increasing doses of venlafaxine, and the other three patients took sodium valproate alone in dose of 500 mg twice a day or successive flunarizine, venlafaxine and pregabalin.

### Ethics

The study has been approved by the Ethics Committee of the Anhui Medical University within which the work was undertaken. All participants gave a written consent.

## Results

Through a 20-month period (from April 2014 to Dec 2015), eight patients with LH attended our Neurological department at the first Hospital of Anhui Medical University. The demographic and clinical features of our eight patients are shown in Table 1. Among the eight patients (Table 1), six was female and two male. They were aged 32–74 years (mean 49.6 ± 15.3 years), and the age at onset of the paroxysmal or continuous head pain ranged from 32 to 73 years (mean 43.63 ± 17.5 years). One patient (patient 7) had personal and family history of migraine headaches. However, there was no temporal connection between the migraine headache and the currently reported head pain in this patient. Other seven patients had no other types of recurrent headaches. Apart from that, past medical history was unremarkable in all patients.

**Table 1:** Demographic and clinical features of eight patients with linear headache (LH)

	Patient no.							
	1	2	3	4	5	6	7	8
Sex	Female	Female	Female	Male	Female	Male	Female	Female
Age (years)	74	54	40	46	46	37	64	32
Age at onset (years)	73	34	20	45	46	35	64	32
Other headaches	None	None	None	None	None	None	Migraine	None
*LH paroxysms*								
Side of LH	Left	Right	Right and left shift	Right	Left	Left	Left	Left
Headache area	Occipital–forehead	Occipital–nasion	Occipital–nasion	Occipital–forehead	Occipitocervical–nasion	Occipitocervical–nasion	Occipitocervical–forehead	Occipital–nasion
Duration^b^	1 h	1 day	1–2 days	Persistent and episodic worsening (1–3 h)	Persistent and episodic worsening (2–3 h)	1 h	2–3 h	Half day
Character	Pressure-like and pulsating	Distending and aching pain	Indescribable	Distending	Pulsating	Pulsating	Pulsating	Distending
Intensity^a^	6–7	7–8	6–7	7–8	6–7	7–9	7–8	7–8
Acompaniments	Nause	None	Awareness of defecation	Ipsilateral temporal mild pain	Diziness and bilateral temporal mild pain	Left temporal mild pain	None	Diziness and nause
Frequency	1/2–3 days	2–3/month to 1/2–3 months	1–2/month	1/day^c^	1/day^c^	1–2/month to 1/day	1/day	1/day
Triggering factors	−	−	Menstruation	−	−	Noise and bright light	Noise, resting after physical activity	Fatigue
Interictal symptoms	None	None	None	−	−	None	None	None
Temporal pattern	Chronic	Chronic	Chronic	Chronic	−	With remission	−	−
Length of symptomatic period	1 year	20 years	20 years	6 months	1 month	2.5 years	1 month	Half month
*Therapy*								
Medicine	Flunarizine and Venlafaxine	Flunarizine and Venlafaxine	Sodium valproate and flunarizine	Sodium valproate and flunarizine; Venlafaxine and pregabalin	Sodium valproate	Flunarizine	Flunarizine	Flunarizine
Response	Recurrence prevented	Frequency and intensity reduced	Non-menstral pain prevented and menstral pain unchanged	LH pain prevented and the Occipitocervical point pain unchanged	Recurrence prevented	Recurrence prevented	Frequency and intensity reduced	Recurrence prevented

− not applicable

^a^Visual Analogical Scale, 0 = no pain; 10 = the worst pain imaginable

^b^Half day, 3–10 h; One day, 10–24 h

^c^This frequency of 1/day means 1 time pain worsening per day

All patients complained of strictly unilateral paroxysmal or continuous head pain which almost invariably recurred on the same side. Five patients had symptoms on the left and two patients on the right, while the attacks shifted sides in only one patient. All patients reported gradual onset of pain among a linear trajectory of 5 mm to 10 mm in width, linking the occipital or occipitocervical region with the ipsilateral nasion or forehead region. No patient reported radiation or moving of the pain along the linear trajectory. The pain attack duration would be 1 h to 2 days in six patients, but persistent for 1–6 months in two patients of whom one had episode of pain worsening for 1–3 h. The pain character was pulsating or distending in most cases, but pressure-like in one patient and aching pain in another one patient, among these two patients, one also had pulsating feature and another one also distending feature sometimes. The pain character was indescribable in one patient. All patients denied that the pain was epicranial but complained it intracranial. During the attack, accompanied nausea without vomiting was reported in two patients, ipsilateral or bilateral dizziness reported in two patients, ipsilateral or bilateral temporal mild pain in three patients and desire to defecate in one patient, while no accompanied symptom reported in two patients. The paroxysms were usually spontaneous in four cases, but triggered by noise and bright light in one case, by noise and resting after physical activity in one case, by fatigue in one case and by menstruation in one case. During the interictal period, six patients had no symptoms at all, the other two had persistent LH pain. The frequency of attacks was variable, ranging from one attack every 2–3 months to one attack per day. The temporal pattern of the attacks was chronic in four patients who had been having attacks for 1–20 years without a remission. One patient had a remission period of 1 year between recurrent periods. The remaining three patients had short symptomatic period of half to 1 month and the pain was persistent or recurrent every day.

With regard to examinations, no skin change was noted along the painful linear trajectory either during the ictal period or during the interictal period in all patients, but a hyperaesthesia to light touch noted along the painful linear trajectory in one patient (patient 2). Regional mild pain was noted in two patients while palpating the greater occipital nerves (patient 2 and 4), but no abnormal sensation noted in all other patients. Neurological examination, brain CT or MRI, and blood tests were normal in all patients. Cervical MRI or X-ray results revealed no special in almost all patients except in one patient (patient 1) who had degenerative changes of cervical vertebra. FRT results were negative in almost all patients except in one patient (patient 1) whose head rotation ranges to both sides were positive (range reduced more than 10° from the normal range of 44°).

Before coming to our department, 5 out of the 8 patients had ever tried analgesics while the headache was attacking. 2 patients (patient 2 and 3) could not remember what the exact analgesics they had ever taken. 2 patients (patient 1 and 5) had ever tried analgesics consisting of aminopyrine, phenacetin, caffeine and phenobarbital, and the pain was alleviated mildly in one patient (patient 1) but not in another one (patient 5). One patient (patient 4) had ever tried indomethacin and the pain was not reduced. All these 5 patients abandoned the medications after trying couple of times. Regarding therapy after they came to our department, eight patients accepted prophylatic treatment and had good responses. Two patients (patient 6 and 8) treated with flunarizine (5 mg twice a day) alone had the headache recurrence prevented and stopped taking medicine after 1 month treatment. Three months later, the headache recurred in one patient (patient 8). Three other patients (patients 1, 2 and 7) also took flunarizine (5 mg twice a day) alone first, and among these three patients, pain frequency and intensity was reduced in one patient (patient 7), but not changed in the other two patients (patients 1 and 2) who were further added with venlafaxine (from 75 mg once a day to 150 mg once a day) with pain recurrence prevented in one patient (patient 1) and pain frequency and intensity reduced in another one patient (patient 2). Sodium valproate (500 mg twice a day) alone relieved the LH pain in one patient (patient 5) but only reduced the pain severity in another one patient (patient 3) who was then added with flunarizine (5 mg twice a day) and had the non-menstral pain prevented but menstral pain unchanged. For the last patient (patient 4), sodium valproate (500 mg twice a day) alone, sodium valproate (500 mg twice a day) together with flunarizine (5 mg twice a day), or venlafaxine (from 75 mg once a day to 150 mg once a day) alone failed to relieve the pain, but venlafaxine (150 mg once a day) in combination with pregabalin (150 mg twice a day) prevented the LH pain with the occipitocervical point pain left there unchanged.

## Discussion

LH is a recently described headache pattern which apparently constitutes a new headache syndrome or a new variant of migraine. Table 2 summarizes the main clinical features of the twenty LH cases that have been described so far: twelve cases in the original description by Wang et al. (2014b) and eight new cases in the present communication. Pareja et al. reported another possible one but the clinical description is not full (Pareja and Bandres 2015). Up to now, there has been a female predominance (1.5:1), and the age at onset has ranged from 20 to 73 years old (mean 41.8). LH is essentially characterized by episodic or chronic unilateral pain distributed in a linear trajectory of 5 mm to 10 mm in width, linking one endpoint in occipital (60 %) or occipitocervical (40 %) region with another endpoint in ipsilateral nose (50 %), forehead (45 %) or parietal (5 %) region, and this linear trajectory is parallel to the sagittal midline of the head. In some patients, the pain is followed by ipsilateral (10 %) or bilateral (5 %) temporal mild pain, nausea with or without vomiting (30 %), dizziness (20 %), or photophobia/phonophobia (5 %). The episodic duration ranges mostly (90 %) from hours to days. Although some patients have a past history of migraine (15 %), there is no temporal relationship between the migraine headache and this newly described head pain. Painful episodes can be triggered by noise, depression, resting after physical activity (5 %), stress, staying up late (5 %) and menstruation (10 %). Pain frequency has varied widely from one to two attacks per month to four to five attacks per day. Two patients (patients 4 and 5 of our series) had persistent head pain for the symptomatic period of 1–6 months, but they also had episodic worsening of head pain within an unaltered distribution in a frequency of one time per day during the persistent symtomatic period.

**Table 2:** Main features of the twenty patients with LH described to date

	Wang et al. (n = 12)	Current series (n = 8)	Total (n = 20)
Female/male ratio	6/6 = 1:1	6/2 = 3:1	12/8 = 1.5:1
Mean age at onset (years)	40.6 ± 12.0	43.63 ± 17.51	41.8 ± 14.12
Age range at onset (years)	20–73	32–74	20–74
Severity of pain	Moderate to severe	Moderate to severe	Moderate to severe
*Pain character*			
Pressure-like	3:12 = 25 %	1:8 = 12.5 %	4:20 = 20 %
Distending/stretching	7:12 = 58.3 %	3:8 = 37.5 %	10:20 = 50 %
Throbbing/pulsating	3:12 = 25 %	4:8 = 50 %	6:20 = 30 %
*Accompaniments*			
Nausea	6:12 = 50 %	2:8 = 25 %	8:20 = 40 %
Diziness	2:12 = 16.7 %	2:8 = 25 %	4:20 = 20 %
Photophobia	0 %	1:8 = 12.5 %	1:20 = 5 %
Phonophobia	0 %	1:8 = 12.5 %	1:20 = 5 %
*Episode duration (range)*			
Less than 2 h	3:12 = 25 %	2:8 = 25 %	7:20 = 35 %
Half day (2–10 h)	5:12 = 41.7 %	3:8 = 37.5 %	7:20 = 35 %
1 day (10–24 h)	4:12 = 33.3 %	1:8 = 12.5 %	6:20 = 30 %
More than 1 day (>24 h)	4:12 = 33.3 %	3:8 = 37.5 %	7:20 = 35 %
Triggers	2:12 = 16.7 %	4:8 = 50 %	6/20 = 30 %

There are several entities to which differential diagnosis need to be considered. EF, a recently described novel headache syndrome, is characterized by brief pain paroxysms starting in a particular area of the posterior scalp, and rapidly radiating forwards along a linear trajectory to reach the ipsilateral forehead, eye, or nose in a few seconds (Pareja et al. 2008; Guerrero et al. 2010; Cuadrado et al. 2013). The linear pain trajectory in EF is similar to that in LH, but LH is dramatically different from the ultrashort duration (less than 10 s) of moving stabbing or electric pain in EF patient. On the other hand, LH may be associated with EF. Pareja and Bandrés recently reported a patient with long-lasting linear interictal pain between the attacks of EF, and this interictal pain is similar to the LH pain (Pareja and Bandres, 2015). Wang et al. have proposed that this linear interictal pain may be LH triggered by EF (Wang et al. 2015), as EF can trigger the attacks of migraine and cluster headache (Jin and Wang, 2013), and LH has many clinical features similar to that of migraine (Wang et al. 2014b). Moreover, the LH pain area is correspondent to the scalp area innervated by the supraorbital nerve (SON) and the greater occipital nerve (GON), we need to differentiate LH from trigeminal (TN) and occipital neuralgia (ON) and some trigeminal autonomic cephalalgias (TACs) such as paroxysmal hemicrania (PH). But the much loner pain duration, the accompaniments of dizziness, and nausea, the triggering factors of noise, bright night, resting after physical activity and menstruation in LH makes it obviously different from TN and ON. PH is typically characterized by repeated attacks of severe, strictly unilateral pain lasting 2–30 min, localized to orbital, supraorbital, and temporal areas accompanied by ipsilateral autonomic features of conjunctival, nasal, facial and eyelid symptoms or signs. But some atypical PH patients do not have accompaniment of autonomic symptoms and the pain duration may extend to hours (Boes and Dodick 2002; Maggioni 2010; Prakash et al. 2013). This atypical episodic or chronic PH may have some LH-like features, but the LH pain striding the trigeminal and occopital nerve innervating areas is obviously different from the PH pain localized to orbital, supraorbital, and temporal areas or in any combination of these sites. These PH pain sites are innervated by trigeminal nerve only, as PH is a type of trigeminal cephalalgia. Thus, LH is different from PH in headache classification.

CEH has rarely been described as pain distributing in a line-shaped area (Cooper et al. 2007) and, in rare cases, it may be accompanied with photophoboa, phonophobia, dizziness or nausea (Hall et al. 2010b). This clinical characteristics are similar to that of LH, thus, we need to differentiate LH from CEH. In fact, there are many different characteristics between LH and CEH. First, the CEH pain attack starts in the neck, eventually spreads to the oculofrontotemporal area (Sjaastad et al. 1990, 1998; Antonaci et al. 2006), but the pain in LH is fixed, and the one endpoint of pain trajectory in LH is not the oculofrontotemporal area but a point in nasion or forehead region, another endpoint is not the neck but a point in occipital or occipitocervical region. Second, CEH has been defined, in principle, as a unilateral headache without sideshift (Antonaci et al. 2006), but the sideshift of pain exists in two out of twenty LH patients (20 %), indicating that the pain sideshift is not uncommon in LH. Third, menstrual-type headaches may uncommonly be cervicogenic (Lieba-Samal and Wober 2011), a study indicated that one out of one hundred (1 %) cases of menstrual-type headaches was cervicogenic headache (Miziara et al. 2003). In our series of LH patients, two out of twenty (10 %) patients had menstrual-type headache, indicating that menstrual-type headache is not uncommonly LH type. Fourth, the FRT was positive only in one out of eight LH patients (12.5 %), but positive in fourteen out of twenty CEH patients (70 %) (Hall et al. 2010b). Hereby, we may conclude that LH is significantly different from CEH, and this is further supported by its well response to flunarizine and sodium valproate which had never been shown effective in CEH. Finally, the literature-reported CEH with pain restricted in a line-shaped area (Cooper et al. 2007) may, in fact, be LH but not CEH, as the exclusive diagnostic measure was anesthetic blockades at upper cervical spinal nerves (Cooper et al. 2007), and the anesthetic blockade of the branch of upper cervical spinal nerves, occipital nerve, was also effective in many other types of headaches including migraine headache (Dilli et al. 2015; Inan et al. 2015).

A special head pain that is defined with topographical criteria, nummular headache (NH), may need to be differentiated with LH, as LH is also defined with topographical criteria. NH was first described by Pareja et al. in 2002 (Pareja et al. 2002). NH is a continuous or intermittent pain which is commonly described as oppressive and felt circumscribed within a rounded or elliptical area, typically 1 cm to 6 cm in diameter (Pareja et al. 2002; IHS 2013). Nevertheless, some NH patients show atypical features resembling a migraine pattern such as episodic pain accompanied with nausea, photophobia and phonophobia (Dai et al. 2013), triggered by or aggravated with physical exercise (Mulero et al. 2013; Baron et al. 2015), or related to menstruation (Robbins and Grosberg, 2010). Some LH patients complained a paroxymal or continuous pain of oppressive, distending or throbbing features with no accompanied symptom, while some other LH patients showed pain features resembling a migraine pattern such as episodic pain accompanied with nausea, photophobia and phonophobia, triggered by or aggravated with physical exercise, or related to menstruation. Given the LH is a special type of NH, the linear pain area may be explained as an extension of a rounded or elliptical pain area of NH. And this hypothesis is seemly supported by reports showing that NH pain can affect multiple rounded areas on the head simutaneously (Cuadrado et al. 2009; Porta-Etessam et al. 2010; Guerrero et al. 2011; Rodriguez et al. 2015). Thus, a connection of series of NH pain areas may form a line-shaped pain area as described in LH, otherwise, the small NH rounded pain area with a diameter of 1 cm or shorter may expand in one direction forming a line-shaped area, 5–10 mm in width as described in LH, or expand in all directions forming a large rounded area, 6–8 cm in diameter as reported in NH (Man et al. 2012; IHS 2013). Whereas, it is hard to explain how the pain affected a line-shaped area parallel to the saggital midline of the head in all LH patients, while the pain area can be localized in any part of the head though mostly in the parietal region in NH patients (Pareja et al. 2012). Thus, it is hard to consider LH as a variant of NH, and on the other hand, the therapeutic responses of LH patients to anti-migraine agents do not suggest that LH presents as a variant of NH either.

The first case of LH pain was reported with accompaniment of ophthalmoplegia successive to the episodic line-shaped head pain which had couple of features similar to that of migraine pattern, such as past history of recurrent migraine attacks, accompaniments of nausea, vomiting, and phonophobia, response to anti-migraine therapy. The presentations of this case are suggestive of a subtype of ophthalmoplegic migraine (OM) (Wang et al. 2014a) though the term of OM had been replaced by recurrent painful ophthalmoplegic neuropathy (RPON) in the 3rd edition of the International Headache Classification (ICHD-3) (IHS 2013). This case of ophthalmoplegic LH indicates that LH is closely related to migraine or probably exists as a variant of migraine. From the current and previous LH case series, plenty of similarities can be found between LH and migraine in clinical features including female to male ratio, pulsating and pressure-like pain quality, moderate to severe pain severity, accompaniments of nausea and dizziness, triggering factors, average episode durations and treatment responses to anti-migraine therapy as summarized in Table 3. Furthermore, menstruation-related headaches are predominately migraine or tension type headache (TTH) but scarcely CGH (Karli et al. 2012). In our current and previous case series, two out of twenty had menstruation-related headache, indicating that menstruation-related headache is not uncommon in LH patients, and our LH headaches are of course not TTH as TTH has never been reported to have pain distribution in a line-shaped area. Further, among the patients of menstrual headaches, 88 % patients are those with migraine without aura (Miziara et al. 2003). Thus, the LH pain is more suggested to be related to migraine. Nevertheless, many differences of great significance also exist between LH and migraine as summarized in Table 3. First, the mean age at onset of LH (41.8 ± 14.12 year old) is obviously older than that of migraine (22 ± 15 year old) and all the LH onset is at adult but the migraine onset commonly at child age. Second, the pain quality of distending or stretching is much more common (50 %) in LH than that in migraine (9.7 %). Third, accompaniment of photophobia or phonophobia is rare in LH (5, 5 %) but common in migraine (51.9, 58.7 %). In fact, the features of the head pain fulfilling the diagnostic criteria for migraine without aura were found only in six patients (patients 2, 4, 6, 9, 11 and 12) of the previous series and in three patients (patients 5, 7 and 8) of the current series, if we treated the unilateral line-shaped head pain as unilateral headache, one of the character for migraine diagnosis (IHS 2013). Thus, LH may represent a new headache syndrome but not a variant of migraine.

**Table 3:** Comparison between Linear Headache (LH) from twenty patients and Migraine from literature in demographic and clinical features

	Migraine (Murtaza et al. 2009)^a^	Linear headache
Mean age at onset (years)	22 ± 15	41.8 ± 14.12
Age range at onset (years)	6–65	20–73
Female/Male ratio	141/65 = 2.16:1	12/8 = 1.5:1
Severity of pain	Moderate to severe (Kelman, 2006)	Moderate to severe
*Pain character*		
Pressure-like	23.8 %	4:20 = 20 %
Distending/stretching	9.7 %	10:20 = 50 %
Throbbing/pulsating	31.6 %	6:20 = 30 %
*Accompaniments*		
Nausea	57.8 %	8:20 = 40 %
Vomiting	32 %	4:20 = 20 %
Diziness	17 %	4:20 = 20 %
Photophobia	51.9 %	1:20 = 5 %
Phonophobia	58.7 %	1:20 = 5 %
*Average episode duration (h)*		
Less than 2 h	23.4 %	7:20 = 35 %
Half day (2–10 h)	41.4 %	7:20 = 35 %
1 day (10–24 h)	14.1 %	6:20 = 30 %
More than 1 day (>24 h)	21.1 %	7:20 = 35 %
*Triggered by*		
Noise, bright night, depression, fatigue and resting after physical activity	Yes	Yes
Stress, staying up late	Yes	Yes
Menstruation	Common (Miziara et al. 2003)	Common
Treatment requirement	Approximate half patients (MacGregor et al. 2003)	Most cases
*Treatment*		
Response to flunarizine	Most patients (Luo et al. 2012)	Common
Response to sodium valproate	Most patients (MacGregor et al. 2003), and recommendation with level A evidence (Silberstein et al. 2012)	Common
Response to carbamazepine	Possibably effective in some patients with no new evidence (Silberstein et al. 2012)	No response
Response to oxcarbazepine	No response (Silberstein et al. 2008)	No response

^a^Apart from the references indicated in migraine column, all other data in this column are from reference (Murtaza et al. 2009)

Regarding the pathogenesis of LH, it is unknown, but a migraine headache mechanism might be employed to comprehend it based on the similarities between LH and migraine in clinical features. In the original publication of LH case series, it is proposed that a line-shaped area of meningeal nociceptors parallel to the superior sagittal sinus (SSS) is more prone to be activated by cortical spreading depression (CSD) due to the sensitization of meningeal nociceptors caused by immuno-vascular interactions (Levy 2012). This results in a line-shaped cephalalgia through migraine pain pathway, as CSD-induced long-lasting activation of meningeal nociceptors innervated by the fibers of SON or GON has been accepted to be one of the original migraine pathophysiological processes leading to the activation of central trigemino-vascular neurons in the spinal trigeminal nucleus (C1-2) underlying migraine headache (Zhang et al. 2010; Zhao and Levy 2015). This proposed pathogenesis related to CSD is seemly supported by the effective responses to anti-migraine drugs of flunarizine, venlafaxine, and anti-epileptic drug of sodium valproate which had been shown able to suppress CSD (Wauquier et al. 1985; Ayata et al. 2006) or reduce depolarization-evoked glutamate release (Musazzi et al. 2010; Milanese et al. 2013), basis of CSD which is a common therapeutic target for currently prescribed migraine prophylactic drugs (Costa et al. 2013). This proposed pathogenesis is further supported by our recent case study of a woman who had a recurrent head pain circumscribed in a coronal line-shaped area around the head, a coronal LH. Similar to the LH pain area close and parallel to the sagittal venous sinus, SSS, this coronal LH pain area is close and parallel to a coronal cambered venous sinus complex, the combination of the confluens of sinus (CFS) and the bilateral cavernous sinus (CS), superior petrosal sinus (SPS) linking the CS with transverse sinus (TS) and TS into which the SPS feeds (Wang et al. 2016), Whereas, this proposed pathogenesis underlying LH is pure speculation, further observations of its clinical characteristics and pathophysiological studies are required to clarify it. For example, studies of the cerebral excitability in LH in comparision with that in migraine without aura in human and studies of venous sinus endophlebitis-induced activation of neurons in the spinal nociceptive center, the trigeminocervical complex, in animal model may be helpful to clarify the pathogenesis of LH. On the other hand, trials of more medications of different pharmacological mechanisms may also be helpful, and this depends on collection of much more cases. In fact, the limitation of our current study report is the small size of case collection and medication trials.

## Conclusions

The current LH cases and those originally described show a clear-cut clinical picture. LH is basically characterized by recurrent or chronic unilateral pain circumscribed in a line-shaped area linking one endpoint in occipital or occipitocervical region with another one endpoint in ipsilateral nose or forehead region, accompanied with or without nausea, vomiting, dizziness and mild temporal pain. So far, anti-migraine therapies have been shown effective in individual patients. This report reinforces the proposal that LH is a new headache syndrome or a new variant of previously known headache syndrome, probably of migraine. Most patients require therapeutic measures. At this stage, no definite explanation can be provided, but the clinical features point to a pathophysiological pathway similar to that of migraine. Further observations and pathophysiological studies are required for a definitive characterization and comprehension of this headache.

## Abbreviations

LH: linear headache
EF: epicranial fugax
CT: computed tomography
MRI: magnetic resonance imaging
CEH: cervicogenic headache
FRT: cervical flexion–rotation test
ICHD: international classification of headache disorders
SON: supraorbital nerve
GON: greater occipital nerve
TN: trigeminal neuralgia
ON: occipital neuralgia
TACs: trigeminal autonomic cephalalgias
PH: paroxysmal hemicrania
NH: nummular headache
OM: ophthalmoplegic migraine
RPON: recurrent painful ophthalmoplegic neuropathy
TTH: tension type headache
SSS: superior sagittal sinus
CSD: cortical spreading depression
CFS: confluens of sinus
CS: cavernous sinus
SPS: superior petrosal sinus
TS: transverse sinus
